# Impact of weight variability on mortality among Korean men and women: a population based study

**DOI:** 10.1038/s41598-019-46037-7

**Published:** 2019-07-02

**Authors:** Daein Choi, Seulggie Choi, Sang Min Park

**Affiliations:** 1 Department of Biomedical Sciences, Seoul National University College of Medicine, Seoul, Korea; 2 Pyeongchang Health Center and County Hospital, Pyeongchang, Korea; 3 Department of Family Medicine, Seoul National University Hospital, Seoul, Korea

**Keywords:** Obesity, Risk factors

## Abstract

The health consequences of weight fluctuation have been controversial and little-studied within Asian populations. We aimed to determine the effect of weight variability on mortality using Korean National Health Insurance Service – National Health Screening Cohort. Weight variability was defined as the average successive variability of body mass index (BMI) of the first (2002 and 2003), second (2004 and 2005), and third (2006 and 2007) health examinations. Then, we used Cox regression models to estimate the effect of weight variability on mortality. Compared to participants within the first quintile (lowest) of weight variability, those within the fifth quintile (highest) had increased the risk of death from all causes (hazard ratio, HR 1.33, 95% confidence interval, CI 1.26–1.41), cardiovascular disease (HR 1.31, 95% CI 1.12–1.53), cancer (HR 1.11, 95% CI 1.02–1.22), and other causes (HR 1.58, 95% CI 1.45–1.73). The risk-increasing effect of weight variability on mortality was preserved after excluding past and current smokers as well as those with pre-existing cardiovascular disease or cancer. In conclusion, high weight variability may lead to elevated risk of death even among healthy never smokers. Therefore, maintaining a steady weight should be recommended to benefit from reduced risk of death.

## Introduction

Obesity is a major risk factor for diabetes, hypertension, dyslipidemia, and cardiovascular diseases^1–3^. Furthermore, numerous studies have reported that obesity is associated with increased risk of mortality^4–7^. However, whether or not weight loss actually leads to health benefits has been controversial. While some studies suggest that weight loss for obese people is associated with reduction in mortality risk^8–11^, other observational studies have shown that weight loss may lead to elevated risk of death^12–14^. Although the reasons for such conflicting results are unclear, it has been suggested that weight fluctuation may play an important role. As individuals who lose weight frequently regain their weight^15,16^, high weight variability may lead to unwanted health outcomes among those who initially lose weight.

After initial studies revealing that high weight variability was associated with increased risk of mortality^17–20^, most recent studies have failed to replicate such results^21–24^. However, most previous studies investigating the effect of weight variability on mortality used relatively small sample sizes within Western populations^17–24^. Although few studies have examined the effect of weight change on mortality^25,26^, only one other study has explored the association between weight fluctuation and mortality within an Asian population^22^. Since Asian people tend to have higher body fat composition compared to Caucasians with similar body mass index (BMI) values^27,28^, the health consequences of weight variability for Asians may be different compared to those within Western populations. Therefore, studies investigating the effect of weight variability on mortality in a large Asian population are needed.

In this population-based longitudinal study, we aimed to determine the effect of weight variability on all-cause and cause-specific mortality among a large population of Korean men and women using the Korean National Health Insurance Service (NHIS) database.

## Methods

### Study population

Since the National Health Insurance Act in 1989, the NHIS provides health insurance for 97% of all Korean citizens^29,30^. Furthermore, the NHIS provides biannual health screening examinations for all citizens aged 40 years or more. During the health examination, participants are required to fill self-reported questionnaires on health behaviors, past history, and family history, as well as undergoing basic physical examinations measuring height, weight, and blood pressure. Furthermore, all participants undergo blood tests to determine various biochemical markers such as fasting serum glucose (FSG) and total cholesterol. Based on the data from health examinations, sociodemographics, hospital use, and death registries, the NHIS constructed a database by a simple random sampling method. The resulting cohort, the National Health Insurance Service – National Health Screening Cohort (NHIS-HEALS), followed up men and women aged 40 years or older who underwent health examinations in 2002 until 2015. The data used in this study is directly available via the NHIS database registration system. Numerous previous studies have used this NHIS-HEALS database for epidemiological studies, and its validity has been described in detail elsewhere^4,31^.

Among 264,480 men and women aged 40 years or older who underwent health examinations during the first (2002–2003), second (2004–2005), and third (2006–2007) periods, we excluded 319 participants with missing BMI values. Furthermore, 22,856 individuals with missing values on covariates and 665 participants who died before the index date of 1 January 2008 were excluded. The final study population consisted of 240,640 men and women.

### Key variables

BMI values, determined by dividing the weight in kilograms by the height in meters squared, were measured for each of the three health examination periods. Then, weight variability was calculated by the average successive variability (ASV) method^32^. In detail, weight variability was determined by calculating the averaged absolute values of the differences in BMI between examinations. Then, the study population was divided into five groups according to quintiles of weight variability, with the first quintile having the lowest weight variability and the fifth quintile having the highest weight variability.

Death registry data from the Korea National Statistical Office merged with the NHIS database were used to determine deaths and causes of death. Death from all causes was identified as participants with death dates between 1 January 2008 and 31 December 2015. Among those with a death date, cause-specific mortality was determined by the cause of death, which identifies the cause of death using the Tenth Revision of International Classification of Diseases (ICD-10) codes by the World Health Organization. Death from cardiovascular disease was defined when the cause of death was due to ischemic heart disease (ICD-10 codes I20–I25) or cerebrovascular disease (I60–69)^33^. Death from cancer was defined when the cause of death was due to cancer (C00–C97). Finally, death from other causes was defined when the cause of death was not due to cardiovascular disease or cancer.

Potential confounding covariates considered were age (continuous, years), sex (categorical, men or women), baseline BMI (continuous, kg/m^2^), change in BMI (continuous, kg/m^2^), household income (categorical, first, second, third, or fourth quartiles), smoking (categorical, never smoker, past smoker, or current smoker), alcohol consumption (categorical, <1, 1–2, 3–4, or 5 times per week), physical activity (categorical, 0, 1–2, 3–4, 5–6, or 7 times per week), systolic blood pressure (continuous, mmHg), FSG (continuous, mg/dL), total cholesterol (continuous, mg/dL), underlying cancer (categorical, yes or no), and underlying cardiovascular disease (categorical, yes or no). BMI change, which was included to take direction of weight change into account, was determined by the difference in BMI values between the third and first health examinations. Household income was determined based on the participant’s insurance premium, among entire Korean population. Finally, underlying cardiovascular disease and cancer were determined when a participant had a record of hospital use with the corresponding ICD-10 codes between 2002 and 2007.

### Statistical analysis

All participants were followed-up from 1 January 2008 and ended at the date of death or 31 December 2015, whichever came first. The risk of death from all causes, cardiovascular disease, cancer, and other causes according to weight variability was determined by calculating the hazard ratios (HRs) and 95% confidence intervals (CIs) using Cox proportional hazards regression analysis. In all analyses, the first quintile of weight variability, those with the lowest weight variability, was considered the reference group. Furthermore, *p* for trend values were calculated in order of increasing weight variability for the risk of death from all causes, cardiovascular disease, cancer, and other causes.

Stratified analyses of the effect of weight variability on death from all causes were conducted according to subgroups of sex, initial BMI, physical activity, and direction of weight change. Initial BMI was grouped into normal weight (<23.0 kg/m^2^), overweight (23.0–24.9 kg/m^2^), and obese (≥25.0 kg/m^2^) according to the World Health Organization Western Pacific Region guideline^34^. For sensitivity analyses, the effect of weight variability on death from all causes was conducted after excluding past and current smokers, as well as individuals with underlying cancer or cardiovascular disease. Also, sensitivity analysis without adjustment for change in BMI was conducted. Additional assessment on the effect of weight change on death from all causes including 79,328 participants who underwent health examinations twice during the first (2002–2003) and the third (2006–2007) periods was done. The participants underwent examinations twice only during the first (2002–2003) and the second (2004–2005) periods were not included in this analysis since the weight variability among these participants were at different time period. For this analysis, we chose stable weight (gain or loss of BMI <2.0 kg/m^2^) as the reference group, and compared the risk of weight gain (gain of BMI ≥2.0 kg/m^2^) and weight loss (loss of BMI ≥2.0 kg/m^2^) group. Subgroup analysis determining the effect of weight change direction and fluctuation on mortality with stratified analyses based on initial BMI was also conducted. This analysis was also conducted by choosing stable weight (gain or loss of BMI <1.0 kg/m^2^ between each examinations) as the reference, and compared to continuous weight gain (gain of weight between every examinations with at least one gain of BMI ≥1.0 kg/m^2^), continuous weight loss (loss of weight between every examinations with at least one loss of BMI ≥1.0 kg/m^2^), and weight fluctuation (other than stable weight, continuous weight gain or continuous weight loss) group. Finally, a linear association between BMI variability and mortality was validated graphically using a Loess line through Martingale residuals.

Statistical significance was defined as a *p* value of less than 0.05 in a two-sided manner. All data collection and statistical analyses were conducted using SAS 9.4 (SAS Institute, Cary, NC, USA).

### Ethical considerations

The Seoul National University Hospital Institutional Review Board approved this study (IRB number: X-1701/378-902). The requirement for informed consent from the participants was waived as the NHIS-HEALS database is anonymized according to strict confidentiality guidelines.

## Results

Table 1 depicts the descriptive characteristics of the study population according to weight variability. Among 240,640 study participants, approximate of 20.1%, 19.8%, 20.0%, 20.1% and 20.0% individuals were allocated for the first, second, third, fourth, and fifth quintiles of weight variability group, respectively. The mean weight variability values determined by ASV for the first, second, third, fourth, and fifth quintile groups were 0.24 kg/m^2^, 0.50 kg/m^2^, 0.73 kg/m^2^, 1.03 kg/m^2^, and 1.91 kg/m^2^, respectively. The proportion of weight gain and weight loss among total study population was 47.5% and 52.5%, respectively. The overall prevalence of obesity at the first and third examination was 34.3% and 33.8%, respectively. Participants who were female, had lower household income, were never smokers, did not consume alcohol, and had underlying cardiovascular disease or cancer tended to have greater weight variability.

**Table 1:** Descriptive characteristics of the study population.

	Weight variability (ASV)					*p* value
	First quintile	Second quintile	Third quintile	Fourth quintile	Fifth quintile	
Number of people	48,385	47,745	48,127	48,307	48,076	
Age, years, mean (SD)	55.4 (8.4)	55.9 (8.6)	56.1 (8.7)	56.6 (9.0)	57.7 (9.5)	<0.001
**Sex**, **N (%)**						
Men	30,804 (63.7)	27,757 (58.1)	28,162 (58.5)	27,595 (57.1)	24,568 (51.1)	<0.001
Women	17,581 (36.3)	19,988 (41.9)	19,965 (41.5)	20,712 (42.9)	23,508 (48.9)	
**Weight variability (ASV)**, **kg/m**^**2**^						
Mean (SD)	0.24 (0.10)	0.50 (0.06)	0.73 (0.07)	1.03 (0.11)	1.91 (1.13)	
Range	0.00–0.38	0.38–0.61	0.61–0.86	0.86–1.24	1.24–47.5	
**Initial body mass index**, **kg/m**^**2**^						
Mean (SD)	23.7 (2.7)	23.7 (2.7)	23.9 (2.8)	24.1 (2.9)	24.6 (3.4)	<0.001
Proportion of ≥25.0 kg/m^2^, N (%)	14,607 (30.2)	14,246 (29.8)	15,955 (33.2)	17,318 (35.9)	20,344 (42.3)	<0.001
**Change in body mass index**, **kg/m**^**2**^						
Mean (SD)	0.01 (0.44)	0.02 (0.80)	0.03 (1.10)	0.01 (1.45)	-0.12 (2.63)	<0.001
Proportion with weight gain, N (%)	21,517 (44.5)	23,329 (48.9)	23,475 (48.8)	23,503 (48.7)	22,430 (46.7)	<0.001
**Household income**, **N (%)**						
First quartile (highest)	20,936 (43.3)	19,309 (40.4)	19,238 (40.0)	18,178 (37.6)	15,988 (33.3)	<0.001
Second quartile	13,598 (28.1)	13,430 (28.1)	13,717 (28.5)	13,981 (28.9)	14,019 (29.2)	
Third quartile	8,522 (17.6)	9,240 (19.4)	9,270 (19.3)	9,780 (20.2)	10,708 (22.3)	
Fourth quartile (lowest)	5,329 (11.0)	5,766 (12.1)	5,902 (12.3)	6,368 (13.2)	7,361 (15.3)	
**Smoking**, **N (%)**						
Never smoker	33,344 (68.9)	34,117 (71.5)	34,312 (71.3)	34,697 (71.8)	35,884 (74.6)	<0.001
Past smoker	4,979 (10.3)	4,375 (9.2)	4,663 (9.7)	4,483 (19.9)	4,067 (8.5)	
Current smoker	10,062 (20.8)	9,253 (19.4)	9,152 (19.0)	9,127 (18.9)	8,125 (16.9)	
**Alcohol consumption**, **times per week**, **N (%)**						
None	26,300 (54.4)	27,654 (57.9)	27,760 (57.7)	28,717 (59.5)	30,727 (63.9)	<0.001
<1	7,963 (16.5)	7,391 (15.5)	7,307 (15.2)	7,016 (14.5)	6,324 (13.2)	
1–2	9,411 (19.5)	8,354 (17.5)	8,532 (17.7)	8,065 (16.7)	6,904 (14.4)	
3–4	3,167 (6.6)	2,910 (6.1)	3,037 (6.3)	2,921 (6.1)	2,574 (5.4)	
≥5	1,544 (3.2)	1,436 (3.0)	1,491 (3.1)	1,588 (3.3)	1,547 (3.2)	
**Physical activity**, **times per week**, **N (%)**						
None	21,333 (44.1)	22,196 (46.5)	22,507 (46.8)	23,220 (48.1)	24,966 (51.9)	<0.001
1–2	15,207 (31.4)	14,382 (30.1)	14,038 (29.2)	13,554 (28.1)	12,101 (25.2)	
3–4	7,023 (14.5)	6,533 (13.7)	6,636 (13.8)	6,370 (13.2)	5,795 (12.1)	
5–6	1,650 (3.4)	1,588 (3.3)	1,662 (3.5)	1,724 (3.6)	1,641 (3.4)	
7	3,172 (6.6)	3,046 (6.4)	3,284 (6.8)	3,439 (7.1)	3,573 (7.4)	
Systolic blood pressure, mmHg, mean (SD)	125.1 (15.8)	125.2 (15.9)	125.6 (16.1)	126.0 (16.1)	126.8 (16.4)	<0.001
Fasting serum glucose, mg/dL, mean (SD)	97.3 (23.1)	97.4 (23.8)	98.0 (24.7)	98.6 (26.2)	99.8 (29.4)	<0.001
Total cholesterol, mg/dL, mean (SD)	198.1 (35.7)	198.2 (36.0)	198.8 (36.5)	199.2 (36.7)	199.3 (37.7)	<0.001
**Underlying disease**, **N (%)**						
Cancer	2,971 (6.1)	2,912 (6.1)	3,038 (6.3)	3,246 (6.7)	3,744 (7.8)	<0.001
Cardiovascular disease	7,648 (15.8)	7,788 (16.3)	8,243 (17.1)	8,750 (18.1)	9,785 (20.4)	<0.001

*p* value calculated by Chi-squared test for categorical variables and analysis of variance for continuous variables.

Acronyms: ASV, average successive variability; SD, standard deviation; N, number of people.

The effect of weight variability on death from all causes, cardiovascular disease, cancer, and other causes are shown in Table 2. Compared to those within the first quintile (lowest) of weight variability, those within the fifth quintile (highest) of weight variability had increased risk of death from all causes (HR 1.33, 95% CI 1.26–1.41), cardiovascular disease (HR 1.31, 95% CI 1.12–1.53), cancer (HR 1.11, 95% CI 1.02–1.22), and other causes (HR 1.58, 95% CI 1.45–1.73). Furthermore, the risk of death from all causes, cardiovascular disease, cancer, and other causes increased upon greater degrees of weight variability (*p* for trends <0.001).

**Table 2:** Effect of weight variability on death from all causes, cardiovascular disease, cancer, and other causes.

	Body mass index variability (ASV)					*p* for trend
	First quintile	Second quintile	Third quintile	Fourth quintile	Fifth quintile	
**Death from all causes**						
Events	1,910	1,949	2,151	2,523	3,248	
Person-years	380,320	374,992	377,304	377,408	372,556	
HR (95% CI)	1.00 (reference)	0.98 (0.91–1.05)	1.07 (1.00–1.14)	1.15 (1.08–1.22)	1.33 (1.26–1.41)	<0.001
**Cardiovascular disease-related death**						
Events	247	263	275	323	470	
Person-years	380,320	374,992	377,304	377,408	372,556	
HR (95% CI)	1.00 (reference)	1.00 (0.84–1.19)	1.01 (0.85–1.20)	1.06 (0.90–1.25)	1.31 (1.12–1.53)	<0.001
**Cancer-related death**						
Events	900	851	908	1060	1,188	
Person-years	380,320	374,992	377,304	377,408	372,556	
HR (95% CI)	1.00 (reference)	0.93 (0.85–1.02)	0.98 (0.90–1.08)	1.06 (0.97–1.16)	1.11 (1.02–1.22)	<0.001
**Death from other causes**						
Events	763	835	968	1,140	1,590	
Person-years	380,320	374,992	377,304	377,408	372,556	
HR (95% CI)	1.00 (reference)	1.04 (0.94–1.14)	1.19 (1.08–1.31)	1.28 (1.17–1.40)	1.58 (1.45–1.73)	<0.001

Hazard ratio calculated by Cox proportional hazards regression analysis after adjustments for age, sex, baseline body mass index, change in body mass index, household income, smoking, alcohol consumption, physical activity, systolic blood pressure, fasting serum glucose, total cholesterol, underlying cancer, and underlying cardiovascular disease.

Acronyms: ASV, average successive variability; HR, hazard ratio; CI, confidence interval.

Table 3 shows the results from stratified analyses of the effect of weight variability on death from all causes according to subgroups of sex, initial BMI, physical activity, and direction of weight change. The risk of death from all causes was increased among those with the greatest weight variability compared to those with the lowest weight variability for both men (HR 1.33, 95% CI 1.24–1.42) and women (HR 1.32, 95% CI 1.18–1.48). When the participants were divided into normal weight, overweight, and obese individuals during the first health examination, those with the greatest weight variability had elevated risk of death from all causes for normal weight (HR 1.37, 95% CI 1.25–1.49), overweight (HR 1.33, 95% CI 1.18–1.49), and obese (HR 1.24, 95% CI 1.12–1.38) participants. Both participants who exercised (HR 1.31, 95% CI 1.22–1.42) and did not exercise (HR 1.36, 95% CI 1.25–1.49) had elevated risk of death from all causes among those within the fifth quintile compared to those within the first quintile of weight variability. Finally, participants with the greatest weight variability had elevated risk of death from all causes for both those who gained weight (HR 1.30, 95% CI 1.20–1.41) and those who lost weight (HR 1.38, 95% CI 1.27–1.49). For all subgroups, the risk of death from all causes increased upon greater weight variability (*p* for trends <0.001).

**Table 3:** Stratified analysis of the effect of weight variability on death from all causes according to subgroups of sex, initial body mass index, physical activity, and direction of weight change.

		Body mass index variability (ASV)					*p* for trend
		First quintile	Second quintile	Third quintile	Fourth quintile	Fifth quintile	
Sex	Men, events	1,483	1,459	1,574	1,789	2,115	
	Person-years	241,188	216,761	219,617	214,259	188,569	
	HR (95% CI)	1.00 (reference)	0.99 (0.92–1.07)	1.07 (0.99–1.15)	1.14 (1.06–1.22)	1.33 (1.24–1.42)	<0.001
	Women, events	427	490	577	734	1,133	
	Person-years	139,132	158,231	157,687	163,149	183,987	
	HR (95% CI)	1.00 (reference)	0.95 (0.84–1.08)	1.05 (0.83–1.19)	1.17 (1.04–1.32)	1.32 (1.18–1.48)	<0.001
Initial body mass index	<23.0 kg/m^2^, events	888	997	981	1,119	1,392	
	Person-years	153,299	154,233	144,679	136,351	119,840	
	HR (95% CI)	1.00 (reference)	1.05 (0.96–1.15)	1.07 (0.97–1.17)	1.16 (1.06–1.26)	1.37 (1.25–1.49)	<0.001
	23.0–24.9 kg/m^2^, events	487	472	574	630	752	
	Person-years	112,017	108,448	107,032	105,085	94,028	
	HR (95% CI)	1.00 (reference)	0.96 (0.84–1.09)	1.13 (0.99–1.27)	1.19 (1.05–1.33)	1.33 (1.18–1.49)	<0.001
	≥25.0 kg/m^2^, events	535	480	596	774	1,104	
	Person-years	115,005	112,311	125,593	135,972	158,688	
	HR (95% CI)	1.00 (reference)	0.89 (0.79–1.01)	1.00 (0.89–1.12)	1.07 (0.96–1.20)	1.24 (1.12–1.38)	<0.001
Physical activity	No, events	1,047	1,136	1,248	1,512	2,104	
	Person-years	166,925	173,562	175,633	180,330	191,917	
	HR (95% CI)	1.00 (reference)	0.99 (0.91–1.07)	1.06 (0.97–1.15)	1.15 (1.07–1.25)	1.31 (1.22–1.42)	<0.001
	Yes, events	863	813	903	1,011	1,144	
	Person-years	213,395	201,430	201,672	197,077	180,639	
	HR (95% CI)	1.00 (reference)	0.98 (0.89–1.07)	1.08 (0.98–1.18)	1.14 (1.04–1.24)	1.36 (1.25–1.49)	<0.001
Direction of weight change	Weight gain, events	974	966	1,055	1,186	1,486	
	Person-years	212,915	198,898	202,270	196,653	186,107	
	HR (95% CI)	1.00 (reference)	1.01 (0.92–1.10)	1.08 (0.99–1.17)	1.12 (1.03–1.22)	1.30 (1.20–1.41)	<0.001
	Weight loss, events	936	983	1,096	1,337	1,762	
	Person-years	167,405	176,093	175,034	180,754	186,449	
	HR (95% CI)	1.00 (reference)	0.96 (0.87–1.05)	1.06 (0.97–1.15)	1.17 (1.07–1.27)	1.38 (1.27–1.49)	<0.001

Hazard ratio calculated by Cox proportional hazards regression analysis after adjustments for age, sex, baseline body mass index, change in body mass index, household income, smoking, alcohol consumption, physical activity, systolic blood pressure, fasting serum glucose, total cholesterol, underlying cancer, and underlying cardiovascular disease.

Acronyms: ASV, average successive variability; HR, hazard ratio; CI, confidence interval.

Results from the sensitivity analyses of the effect of weight variability on mortality after excluding past and current smokers, as well as participants with underlying cancer or cardiovascular disease are depicted in Table 4. Compared to never smoking healthy participants with the lowest weight variability, those with the greatest weight variability had elevated risk of death from all-causes (HR 1.32, 95% CI 1.19–1.29), cardiovascular disease (HR 1.40, 95% CI 1.06–1.84), and other causes (HR 1.55, 95% CI 1.35–1.79). Furthermore, increasing levels of weight variability was associated with increased risk of death from all causes (*p* for trend <0.001), cardiovascular disease (*p* for trend 0.021), and other causes (*p* for trend <0.001).

**Table 4:** Sensitivity analysis of the effect of weight variability on death from all causes after excluding past and current smokers, and individuals with underlying cancer or cardiovascular disease and current smokers.

	Body mass index variability (ASV)					*p* for trend
	First quintile	Second quintile	Third quintile	Fourth quintile	Fifth quintile	
**Death from all causes**						
Events	797	812	883	1,038	1,232	
Person-years	237,164	237,162	236,873	233,672	228,408	
HR (95% CI)	1.00 (reference)	0.99 (0.90–1.09)	1.06 (0.97–1.17)	1.17 (1.06–1.28)	1.30 (1.19–1.42)	<0.001
**Cardiovascular disease-related death**						
Events	85	97	107	115	170	
Person-years	237,164	237,162	236,873	233,672	228,408	
HR (95% CI)	1.00 (reference)	1.08 (0.81–1.45)	1.14 (0.86–1.52)	1.12 (0.85–1.49)	1.46 (1.12–1.89)	0.005
**Cancer-related death**						
Events	374	334	384	437	439	
Person-years	237,164	237,162	236,873	233,672	228,408	
HR (95% CI)	1.00 (reference)	0.88 (0.76–1.02)	1.00 (0.87–1.16)	1.08 (0.94–1.25)	1.05 (0.92–1.21)	0.055
**Death from other causes**						
Events	338	381	392	486	623	
Person-years	237,164	237,162	236,873	233,672	228,408	
HR (95% CI)	1.00 (reference)	1.09 (0.94–1.26)	1.11 (0.96–1.28)	1.27 (1.11–1.46)	1.50 (1.31–1.72)	<0.001

Hazard ratio calculated by Cox proportional hazards regression analysis after adjustments for age, sex, baseline body mass index, change in body mass index, household income, alcohol consumption, physical activity, systolic blood pressure, fasting serum glucose, and total cholesterol.

Acronyms: ASV, average successive variability; HR, hazard ratio; CI, confidence interval.

Sensitivity analysis without adjustment for change in BMI is depicted in Supplemental Table 1. High weight variability was also associated with the increased risk of death in all cause-specific subgroups. Supplemental Table 2 shows the effect of weight change on death from all causes in inclusion of participants who underwent health examinations twice. Both weight gain and loss were associated with significant increased risk of death, compared to stable weight group. Finally, the effect of weight change direction and fluctuation on mortality is shown in Supplemental Table 3. Compared to stable weight groups, all three of continuous weight gain, continuous weight loss, and weight fluctuation groups were associated with increased risk of all-cause mortality.

## Discussion

In this population-based study of more than 240,000 Korean men and women, we have shown that weight variability is associated with increased risk of death from all causes, cardiovascular disease, and cancer. We also demonstrated that increased risk of death among those with high weight variability was preserved regardless of initial BMI or direction of weight change. To our knowledge, this is the first study to show that those with high weight variability had elevated risk of mortality among never smokers without pre-existing cardiovascular disease or cancer.

Previous studies investigating the association between weight fluctuation and mortality have been controversial. Although initial studies have reported that weight variability is associated with higher risk of mortality^17–20^, numerous subsequent studies have failed to replicate these results^21–24^. Previous studies that failed to show that high weight variability was associated with increased risk of death tended to have smaller study populations (largest being 122,638 people)^24^, shorter follow-up durations, or measured weight variability based on self-reported questionnaires^23,24^. Such limitations may have led to the lack of a significant association between weight variability and mortality in previous studies. However, the exact reasons for the lack of consistency between studies are not yet clear and merit further investigation.

Most recently, a study investigating the effect of weight variability on cardiovascular disease and mortality among coronary artery disease patients revealed that increased weight variability was associated with elevated risk of mortality^32^. This study, however, only contained patients with histories of coronary artery disease^32^, thereby limiting the generalizability of the results. Furthermore, the observational period of weight variability and follow-up period for mortality overlapped^32^, thus leaving the possibility of reverse-causality open. Our results further extend the knowledge of this previous study by demonstrating that high weight variability led to increased risk of death among a large general population.

The association between weight variability and death is even less investigated among Asian population. Recent two studies suggested higher mortality in either moderate weight gain or loss, implying importance of maintaining stable weight^25,26^. However, these studies were based only two weight measurement, thus could not represent exact weight fluctuation^25,26^. There was one other study suggested that weight gain was associated with a reduced risk of mortality^35^. However, the period of determination of weight variability and detection for mortality are overlapped in this study, thus subject to possible reverse causation. To our knowledge, only one other study investigated the association between weight fluctuation and mortality within an Asian population^22^. The study determined that while high weight variability was associated with elevated risk of death among 6,537 Japanese-American men, the positive association was not preserved when limiting the population to never smokers without known pre-existing conditions^22^. The authors concluded that weight variability itself may not elevate the risk of death, but rather that smoking and pre-existing conditions are more important contributors to increasing mortality^22^. However, the results of our study show that increased risk of death due to high weight variability was preserved even among never smokers without pre-existing cardiovascular disease or cancer. Therefore, our results altogether with recent studies suggest that weight variability itself may be a contributor to raising the risk of death while maintaining steady weight could be beneficial^25,26^.

One of the notable findings among Asian studies including our study was that the absolute risk for those who were not obese was higher than those who were overweight^36^, while in studies on Western population showed the magnitude of the absolute risk was lower in normal weight group. Perhaps the different ethnicity, as well as the differing BMI cut-off values according to ethnicity may be the reason behind the difference. Weight reduction may not necessarily be entirely beneficial for overweight individuals and future studies aimed at the benefits of weight reduction among overweight and obese individuals within an Asian population are needed.

Various studies have shown that high weight variability is associated with increased risk of diabetes^32,37^, elevated coronary calcification^38^, cardiovascular-r1elated biomarkers such as insulin and triglycerides^39^, and the net gain of fat mass^40^. Each of these outcomes has been established as risk factors for cardiovascular disease, which could lead to elevated risk of cardiovascular disease^19,32^ and death from cardiovascular disease^41–45^. Furthermore, a previous study has demonstrated that weight fluctuation is associated with shorter telomere length^46^. Shortened telomere length, along with elevated insulin levels, may lead to increased risk of incident cancer, consequently resulting in elevated risk of death from cancer among those with high weight fluctuation. However, the exact mechanisms of how weight variability may elevate the risk of death are unclear and merit further investigation.

Several limitations need to be considered when interpreting the results of our study. First, as weight fluctuation was not determined after the third health examination, possible weight changes after the index date were not accounted for. Second, although BMI is a widely-used measure of adiposity, the exact changes in fat mass according to weight variability could not be measured. Future studies investigating the effect of fat mass and lean mass variability on mortality are needed. Third, the study population consisted of those who participated more than three health examinations, a group that could have certain sociodemographic tendencies. However, we attempted to account for this by adjusting for factors such as household income. We have also conducted a sensitivity analysis by including participant who underwent health examinations twice. Although there was a similar tendency of increased mortality according to weight changes, further studies would be needed to address concerning demographic tendency. Fourth, potential mechanism or cause-specific death other than cancer and cardiovascular disease were not specified. Since the association of weight variability with death in our study was strongest among deaths from other causes, future studies to uncover specific mechanisms and disease associated with high weight variability are needed. Lastly, we could not assess the intentionality of weight change. However, we attempted to take this into account by adjusting for and conducting subgroup analysis according to physical activity, which may be a surrogate marker for intentional weight loss. Furthermore, upon stratified analysis according to the direction of weight change, both individuals who lost and gained weight had elevated risk of death among those with high weight variability.

To our knowledge, this is the first study to demonstrate that high weight variability is associated with increased risk of death among healthy never smokers within a general population. We used a large study population of a nationally representative cohort with a wide range of potential confounding covariates to enhance the reliability and generalizability of our results. Particularly, subgroup analyses and various sensitivity analyses indicated maintaining stable weight would be beneficial. Although The results of our study implicate that maintaining steady weight could be beneficial, determining the risk and benefit of absolute weight change versus weight variability among individual who already gained weight is still difficult issue, thus merit further investigation.

In conclusion, high weight variability may lead to increased risk of death from all causes, cardiovascular disease, and cancer. The detrimental effect of weight fluctuation on elevated mortality risk may be preserved among healthy never smokers regardless of initial BMI, physical activity, and direction of weight change. Maintaining a steady weight should be recommended to benefit from reduced risk of death.

## Supplementary information

Impact of weight variability on mortality among Korean men and women: a population based study
