# Supplementary material for: Impact of weight variability on mortality among Korean men and women: a population based study
Source: Sci Rep. 2019 Jul 2;9:9543. doi: 10.1038/s41598-019-46037-7 (PMC6606749; doi:10.1038/s41598-019-46037-7)
Supplement: Supplementary file 1 — Impact of weight variability on mortality among Korean men and women: a population based study [file 41598_2019_46037_MOESM1_ESM.docx]

**[Title Page for Supplementary Information]**

**Impact of weight variability on mortality among Korean men and women: a population based study**

Daein Choi, MD^1,2^; Seulggie Choi, MD^1^; Sang Min Park, MD, PhD, MPH^1,3^

^1^Department of Biomedical Sciences, Seoul National University College of Medicine, Seoul, Korea

^2^Pyeongchang Health Center and County Hospital, Pyeongchang, Korea

^3^Department of Family Medicine, Seoul National University Hospital, Seoul, Korea

Equal contributors: Daein Choi and Seulggie Choi

Correspondence to:

Sang Min Park, Departments of Family Medicine and Biomedical Sciences, Seoul National University College of Medicine, 101 Daehak-ro, Jongno-gu, Seoul, Korea

Telephone: 82-2-2072-3331

Fax: 82-2-766-3276

E-mail: smpark.snuh@gmail.com

**Supplemental Table 1.** Sensitivity analysis on the effect of weight variability on mortality without adjustment for change in body mass index.

|  | **Body mass index variability (ASV)** | | | | | ***p* for trend** |
| --- | --- | --- | --- | --- | --- | --- |
|  | **First quintile** | **Second quintile** | **Third quintile** | **Fourth quintile** | **Fifth quintile** |  |
| Death from all causes |  |  |  |  |  |  |
| Events | 1,910 | 1,949 | 2,151 | 2,523 | 3,248 |  |
| Person-years | 380,320 | 374,992 | 377,304 | 377,408 | 372,556 |  |
| HR (95% CI) | 1.00 (reference) | 0.98 (0.92-1.05) | 1.07 (1.00-1.14) | 1.15 (1.08-1.22) | 1.34 (1.27-1.42) | <0.001 |
| Cardiovascular disease-related death |  |  |  |  |  |  |
| Events | 247 | 263 | 275 | 323 | 470 |  |
| Person-years | 380,320 | 374,992 | 377,304 | 377,408 | 372,556 |  |
| HR (95% CI) | 1.00 (reference) | 1.00 (0.84-1.19) | 1.01 (0.85-1.20) | 1.06 (0.90-1.25) | 1.31 (1.12-1.54) | <0.001 |
| Cancer-related death |  |  |  |  |  |  |
| Events | 900 | 851 | 908 | 1060 | 1,188 |  |
| Person-years | 380,320 | 374,992 | 377,304 | 377,408 | 372,556 |  |
| HR (95% CI) | 1.00 (reference) | 0.93 (0.85-1.03) | 0.98 (0.90-1.08) | 1.06 (0.97-1.16) | 1.12 (1.02-1.22) | <0.001 |
| Death from other causes |  |  |  |  |  |  |
| Events | 763 | 835 | 968 | 1,140 | 1,590 |  |
| Person-years | 380,320 | 374,992 | 377,304 | 377,408 | 372,556 |  |
| HR (95% CI) | 1.00 (reference) | 1.04 (0.94-1.15) | 1.19 (1.08-1.31) | 1.28 (1.17-1.40) | 1.60 (1.46-1.74) | <0.001 |

Hazard ratio calculated by Cox proportional hazards regression analysis after adjustments for age, sex, baseline body mass index, household income, smoking, alcohol consumption, physical activity, systolic blood pressure, fasting serum glucose, total cholesterol, underlying cancer, and underlying cardiovascular disease.

Acronyms: ASV, average successive variability; HR, hazard ratio; CI, confidence interval.

**Supplemental Table 2.** Sensitivity analysis on the effect of weight change on all-cause mortality among participants who underwent health examinations twice during 2002-2007.

|  | **Stable weight** | **Weight gain** | **Weight loss** |
| --- | --- | --- | --- |
| Participants with 3 health examinations |  |  |  |
| Events | 9,728 | 839 | 1,214 |
| Person-years | 1,663,531 | 106,181 | 112,868 |
| HR (95% CI) | 1.00 (reference) | 1.09 (1.01-1.17) | 1.55 (1.46-1.65) |
| Participants with 2 health examinations |  |  |  |
| Events | 4,276 | 478 | 703 |
| Person-years | 527,868 | 41,617 | 44,833 |
| HR (95% CI) | 1.00 (reference) | 1.18 (1.07-1.30) | 1.64 (1.51-1.78) |

For participants with 3 health examinations, weight change was calculated as the difference between body mass index values between the third and first health examination periods.

Stable weight defined as those who had less than 2.0 kg/m^2^ change in body mass index between the two health examination periods.

Weight gain defined as those who had 2.0 kg/m^2^ or more gain in body mass index between the two health examination periods.

Weight loss defined as those who had 2.0 kg/m^2^ or more loss in body mass index between the two health examination periods.

Hazard ratio calculated by Cox proportional hazards regression analysis after adjustments for age, sex, baseline body mass index, household income, smoking, alcohol consumption, physical activity, systolic blood pressure, fasting serum glucose, total cholesterol, underlying cancer, and underlying cardiovascular disease.

Acronyms: HR, hazard ratio; CI, confidence interval.

**Supplemental Table 3.** Effect of weight change and fluctuation on all-cause mortality.

|  | **Stable weight** | **Continuous weight gain** | **Continuous weight loss** | **Weight fluctuation** |
| --- | --- | --- | --- | --- |
| Overall |  |  |  |  |
| Events | 4,614 | 1,169 | 1,571 | 4,427 |
| Person-years | 893,550 | 184,001 | 179,307 | 625,721 |
| HR (95% CI) | 1.00 (reference) | 1.24 (1.15-1.33) | 1.24 (1.16-1.33) | 1.18 (1.13-1.23) |
| Baseline BMI<23.0 kg/m^2^ |  |  |  |  |
| Events | 609 | 166 | 199 | 604 |
| Person-years | 893,550 | 184,001 | 179,307 | 625,721 |
| HR (95% CI) | 1.00 (reference) | 1.22 (1.01-1.38) | 1.13 (0.95-1.36) | 1.14 (1.01-1.27) |
| Baseline BMI 23.0-24.9 kg/m^2^ |  |  |  |  |
| Events | 2,059 | 477 | 581 | 1,790 |
| Person-years | 893,550 | 184,001 | 179,307 | 625,721 |
| HR (95% CI) | 1.00 (reference) | 1.13 (1.01-1.26) | 1.09 (0.98-1.21) | 1.10 (1.03-1.17) |
| Baseline BMI≥25.0 kg/m^2^ |  |  |  |  |
| Events | 1,946 | 526 | 791 | 2,033 |
| Person-years | 893,550 | 184,001 | 179,307 | 625,721 |
| HR (95% CI) | 1.00 (reference) | 1.34 (1.21-1.49) | 1.44 (1.30-1.58) | 1.27 (1.19-1.35) |

Stable weight defined as those who had less than 1.0 kg/m^2^ change in body mass index between the first and second, as well as the second and third health examination periods.

Continuous weight gain defined as those who were not included in the no weight change group and had increasing body mass index values during subsequent health examinations.

Continuous weight loss defined as those who were not included in the no weight change group and had decreasing body mass index values during subsequent health examinations.

Weight fluctuation defined as those who were not included in the stable weight group and had either weight gain followed by weight loss or weight loss followed by weight gain.

Hazard ratio calculated by Cox proportional hazards regression analysis after adjustments for age, sex, baseline body mass index, household income, smoking, alcohol consumption, physical activity, systolic blood pressure, fasting serum glucose, total cholesterol, underlying cancer, and underlying cardiovascular disease.

Acronyms: HR, hazard ratio; CI, confidence interval; BMI, body mass index.
